# SLC34A2 Targets in Calcium/Phosphorus Homeostasis of Mammary Gland and Involvement in Development of Clinical Mastitis in Dairy Cows

**DOI:** 10.3390/ani14091275

**Published:** 2024-04-24

**Authors:** Xueying Wang, Bohao Zhang, Weitao Dong, Yu Zhao, Xingxu Zhao, Yong Zhang, Quanwei Zhang

**Affiliations:** 1 College of Veterinary Medicine, Gansu Agricultural University, Lanzhou 730070, China; wangxydd2018@163.com (X.W.); zhangbhgs@163.com (B.Z.); d.wt2008@163.com (W.D.); zhaoxx@gsau.edu.cn (X.Z.); 2 Gansu Key Laboratory of Animal Generational Physiology and Reproductive Regulation, Lanzhou 730070, China; 3 College of Life Science and Biotechnology, Gansu Agricultural University, Lanzhou 730070, China; yzhao@gsau.edu.cn

**Keywords:** mastitis, SLC34A2, calcium/phosphorus homeostasis, proteomics

## Abstract

**Simple Summary:**

Unbalanced Ca/P metabolism can induce mastitis in dairy cows, and solute carrier family 34 member 2 (SLC34A2) can affect transcellular inorganic phosphate absorption. In this study, the expression and localization of SLC34A2 in the mammary gland tissue of cows were detected and analyzed. In addition, based on the data-independent acquisition (DIA) proteomics data, we identified 12 critical differentially expressed proteins (DEPs) included in 11 Gene Ontology (GO) terms and two pathways that interacted with SLC34A2 using bioinformatics analysis. The results revealed that SLC34A2 and DEPs could regulate Ca/P metabolism and homeostasis and participate in the occurrence and development of clinical mastitis in dairy cows. These results provide a basis for understanding SLC34A2’s function in Ca/P metabolism and the pathological mechanism of cow mastitis.

**Abstract:**

The type II Na/Pi co-transporter (NaPi2b), encoded by the solute carrier (SLC) transporter 34A2 (SLC34A2), is responsible for calcium (Ca) and phosphorus (P) homeostasis. Unbalanced Ca/P metabolism induces mastitis in dairy cows. However, the specific role of SLC34A2 in regulating this imbalance in Holstein cows with clinical mastitis (CM) remains unclear. The aim of this study was to investigate the role of SLC34A2 and identify differentially expressed proteins (DEPs) that interact with SLC34A2 and are associated with Ca/P metabolism in dairy cows with CM. Immunohistochemical and immunofluorescence staining results showed that SLC34A2 was located primarily in the mammary epithelial cells of the mammary alveoli in both the control (healthy cows, Con/C) and CM groups. Compared to the Con/C group, the relative expression of the *SLC34A2* gene and protein were significantly downregulated in the CM group. We identified 12 important DEPs included in 11 GO terms and two pathways interacting with SLC34A2 using data-independent acquisition proteomics. The PPI (protein-and-protein interaction) network results suggested that these DEPs were associated with ion metabolism and homeostasis, especially SLC34A2. These results demonstrate that SLC34A2 downregulation is negatively correlated with the occurrence and development of CM in Holstein cows, providing a basis for exploring the function and regulatory mechanism of SLC34A2 in Ca/P metabolism and homeostasis in Holstein cows with CM.

## 1. Introduction

Mastitis, an inflammatory condition of the mammary gland (MG), seriously affects milk production and quality, delays the postpartum estrus cycle and gestation, especially in cows with clinical mastitis (CM), and results in significant economic losses in the dairy industry. CM is induced by multiple factors, such as physicochemical stimuli, pathological infection, and immunological dissonance [1]. With these factors in mind, researchers have made substantial efforts to prevent and treat mastitis [2,3], achieving significant breakthroughs. However, mastitis, particularly CM, remains a prevalent and costly disease for the dairy industry. Consequently, undiscovered or overlooked pathogens implicated in CM in dairy cows require further attention.

Macrominerals, including calcium (Ca) and phosphorus (P), are essential for numerous physiological functions, such as supporting the immune system, facilitating bone growth and development, enabling myocyte contraction, and regulating papillary sphincter constriction and papillary duct closure [4,5]. For example, hypocalcemia [6], which is caused by an imbalance between the inflow and outflow of the extracellular Ca pool, causes mastitis due to abnormal papillary sphincter constriction and papillary duct closure. Postparturient hypophosphatemia is defined as a subnormal plasma inorganic P concentration during the perinatal period in dairy cows, which might cause hypocalcemia or mastitis [7]. Previous studies [8,9] also suggest that unbalanced ion homeostasis, particularly in the Ca/P ratio, and abnormal dynamic changes in Ca/P contents in the serum are also important factors that induce mastitis. Maintaining a normal Ca/P content is of great significance to ion homeostasis in healthy dairy cows. However, the regulatory mechanisms and relationships involved in CM occurrence and the development of Ca-P metabolism in dairy cows remain unknown.

In mammals, most P forms hydroxyapatite crystals with Ca. Enhanced availability and absorption of P cause P overload and negatively affect Ca metabolism [10]. Solute carrier (SLC) transporters, the largest family of transmembrane proteins, facilitate the exchange of various substances, including endogenous and xenobiotic compounds, using an electrochemical or ion gradient [11]. Type II Na/Pi co-transporter (NaPi2b), a multitransmembrane sodium-dependent phosphate transporter, is encoded by solute carrier family 34 member 2 (SLC34A2), which is responsible for transcellular inorganic phosphate absorption [12,13]. Previous studies have shown that SLC34A2 is widely expressed in multiple organs and plays a critical role in several malignant tumors [14,15]. Depletion of SLC34A2/NaPi-IIb causes perinatal mortality in rats [11]. This finding suggests that SLC34A2 is closely associated with perinatal diseases. However, studies describing the expression, distribution, and function of SLC34A2 in dairy cows with CM are limited. The aim of this study was to investigate the role of SLC34A2 and identify differentially expressed proteins (DEPs) that interact with SLC34A2 and are associated with Ca/P metabolism in healthy dairy cows or those with CM based on our previous data-independent acquisition (DIA) proteomics data. The changes and relationships of SLC34A2 are also evaluated in the MGs of healthy or CM dairy cows, which may contribute to a better understanding of SLC34A2’s function in Ca/P homeostasis and its pathogenesis in dairy cows with CM.

## 2. Materials and Methods

### 2.1. Sample Preparation and Collection

The Holstein cows (aged 6–7 years old) were undergoing 3rd to 5th lactation with normal metabolism and were not affected by other diseases. Lactating Holstein cows were diagnosed using veterinary udder examination, somatic cell count (SCC), and the Lanzhou Mastitis Test (LMT), as previously described [2,3,16]. Fresh mammary glands (MGs) from healthy Holstein cows (control group, CON/C, *n* = 3) or clinical mastitis cows (experiment group, CM, *n* = 3) were collected after slaughter at a commercial farm in Wuzhong City, Ningxia Province, China, as previously described [1,3,17]. The tissues were stored in liquid nitrogen or fixed with 4% paraformaldehyde immediately. This study was approved by the Animal Ethics Committee of Gansu Agriculture University, Lanzhou, China (NO. GSAU-AEW-2018-0128).

### 2.2. Immunohistochemistry (IHC) Staining

The fixed tissues were embedded in paraffin (Servicebio, Wuhan, China) and cut into 5 μm sections using a microtome (Leica, Weztlar, Germany). Location analysis of SLC34A2 in the mammary gland tissues was performed as previously described [3,16,17]. Briefly, the sections were deparaffinized in xylene and rehydrated. IHC staining was performed using the standard avidin–biotin–peroxidase complex method (ABC staining; Solarbio, Beijing, China) as previously described [2,17]. SLC34A2 antibodies were used at a dilution of 1:250 (Bioss, Beijing, China), and PBS was used instead of the primary antibodies in the negative Con/C group. The slides were observed and photographed under an Olympus BX43 microscope (Olympus, Tokyo, Japan).

### 2.3. Immunofluorescence (IF) Staining

Co-localization analysis of SLC34A2 and cytokeratin18 (CK18, an epithelial cell marker) proteins in the MGs of the Con/C and CM groups was performed as previously described [3,16,17]. Briefly, the antigen-retrieved sections were incubated with primary antibodies (SLC34A2 and CK18 at a dilution ratio of 1:100) and then labeled with different colors of immunoglobulin G as described previously [1]. Nuclei were localized with 4,6-diamidino-2-phenylindole (DAPI; Solarbio, Beijing, China). Images were captured using a fluorescence microscope (Echo Laboratories, San Diego, CA, USA). All immunostaining assays were performed at least in triplicate.

### 2.4. RNA Isolation, cDNA Synthesis and qRT-PCR

Total RNA was isolated from the MGs of the Con/C and CM groups using a FastPure tissue RNA isolation kit (Vazyme, Nanjing, China) according to the manufacturer’s instructions. After quantification and an RNA integrity test, 1 μg of total RNA was applied to reverse transcription to obtain single-stranded cDNA using an Evo M-MLV Kit (AG, Hunan, China). The relative expression levels of SLC34A2 mRNA in MGs of the Con/C and CM groups were determined using a LightCycler 96 real-time PCR system (Roche, Switzerland) based on the manufacturer’s instructions. The primer sequences were as follows: SLC34A2 (NM_174661.2) forward primer CAGTCTGCCAAGCCTGAGAA; and reverse primer, CTCTCTGACCACTTGAGCCC. PCR procedures, including RNA isolation, cDNA synthesis, primer design, and data calculation, were performed as previously described [1,3]. β-actin (NM_173979) forward primer and reverse primer CCAAGGCCAACCGTGAGAA; R, CCAGAGGCATACAGGGACAG) were used as an endogenous control. All qRT-PCR assays were performed at least in triplicate.

### 2.5. Western Blot

Total proteins were extracted from the MGs (50 mg of each sample) of the Con/C and CM using an RIPA reagent (Solarbio, Beijing, China) according to the manufacturer’s instructions. The procedures were performed as previously described [2,3]. Briefly, after quantification using a BCA (Bicinchoninic Acid) kit, total proteins (50 μg) were electrophoresed on a sodium dodecyl sulfate–polyacrylamide gel (SDS-PAGE) and were transferred onto a PVDF (polyvinylidene fluoride) membrane (Millipore CAT Billerica, MA, USA) and blocked at room temperature. The primary antibodies (SLC34A2 and β-actin) were incubated in a 1:1000 dilution ratio at 4 °C overnight. The integrated optical density (IOD) values of each band was quantified using Image-Pro Plus 6.0 software (Media Cybernetics Co., Rockville, MD, USA). β-actin was used as an endogenous control. All blot assays were performed at least in triplicate.

### 2.6. Bioinformatics Analysis

To investigate the biological function and signal transduction of SLC34A2 in the MGs of the Con/C and CM groups, the DIA proteomic sequence data with accession number IPX0003382000/PXD028100 in the ProteomeXchange database were used for Gene Ontology (GO) terms, Kyoto Encyclopedia of Genes and Genomes (KEGG) pathway enrichment analysis, and further identification of the candidate differentially expressed proteins (DEPs) associated with the SLC34A2 protein. In the present study, significant differences in GO terms (*p* < 0.05, *p.adjust* < 0.05), including biological processes (BPs), molecular functions (MFs), and cellular components (CCs), were selected as candidate DEPs interacting with SLC34A2. Heat maps, enrichment circus graphs, volcano plots, and Venn diagrams were drawn using the OmicShare online platform (http://www.omicshare.com/tools, accessed on 20 December 2023) [1,2,3]. Protein-and-protein interaction (PPI), GO, and KEGG pathway networks of these DEPs were constructed using STRING 11.5 and Cytoscape 3.7.1 (IPA, Ingenuity Systems, www.ingenuity.com, accessed on 15 February 2024), including Clue Go and ingenuity pathway analysis [1,3,18]. 

### 2.7. Statistical Analysis

All data are presented as mean ± SD, unless otherwise indicated. Statistical analyses were performed using SPSS 23.0 (SPSS Inc., Chicago, IL, USA). All graphs were constructed using GraphPad Prism 8.0 (GraphPad Software Inc., San Diego, CA, USA) and Adobe Illustrator (Adobe Software Inc., San Jose, CA, USA). The data were analyzed using Student’s *t*-test (between two groups) or one-way analysis of variance (within multiple groups). Statistical significance was set at *p* < 0.05.

## 3. Results

### 3.1. Subcellular Location Analysis of SLC34A2 Protein in the MGs

**Figure 1:**
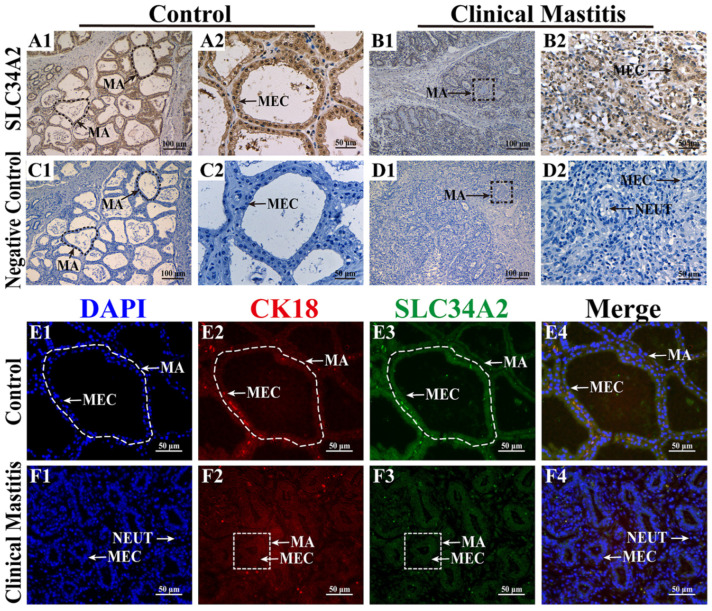
Distribution and co-localization analysis of SLC34A2 protein in MGs. (**A1**–**B2**) IHC staining shows SLC34A2 distribution in MGs of Con/C (**A1**,**A2**) and CM (**B1**,**B2**) groups. (**C1**–**D2**) SLC34A2 negative controls for Con/C (**C1**,**C2**) and CM (**D1**,**D2**) groups. (**E1**–**F2**) Localization in MGs: nuclei (blue, **E1**,**F1**), CK18 (red, **E2**,**F2**), SLC34A2 (green, **E3**,**F3**), with merged CK18 and SLC34A2 (**E4**,**F4**) for Con/C and CM, respectively. Con/C represents healthy Holstein cows; CM indicates cows with clinical mastitis. NC: negative control; MA: mammary alveoli; MECs: mammary epithelial cells; NEUT: neutrophil. Scale bars: 50 nm (200× magnification) and 100 nm (400× magnification).

Subcellular localization and expression of the SLC34A2 protein in the MGs of Holstein cows with Con/C or CM were observed using IHC and IF staining (Figure 1). Positive staining for the SLC34A2 protein was observed in the MGs of the two groups with different degrees of staining (Figure 1A1–B2). In the Con/C group, strong positive expression of the SLC34A2 protein was distributed mainly in the cytoplasm of mammary epithelial cells (MECs) that were neatly arranged in the mammary alveoli (MA) and had a single-cubic or columnar shape (Figure 1A1,A2). In the CM group, positive expression of the SLC34A2 protein was distributed mainly in the cytoplasm of scattered MECs in the MA, which exhibited small alveolar luminal areas with inflammatory cells (Figure 1B1,B2). The negative control group showed no staining for SLC34A2 (Figure 1C1–D2). DAPI staining showed that MECs were arranged neatly in the MA of the Con/C group with intact alveolar luminal areas, whereas they were scattered in the MA of the CM group with intact alveolar luminal areas (Figure 1E1,F1). IF signals of CK18 and SLC34A2 proteins were positive in the MA of both groups (Figure 1E2,E3,F2,F3). Co-localization analysis suggested that SLC34A2 and CK18 were present in the cytoplasm of MECs, especially in the intact alveoli of the Con/C group (Figure 1E4,F4). These results reveal that SLC34A2 plays a crucial role in MECs and is negatively associated with the occurrence and development of CM in Holstein cows.

### 3.2. Expression Patterns of SLC34A2 mRNA and Protein in MGs

**Figure 2:**
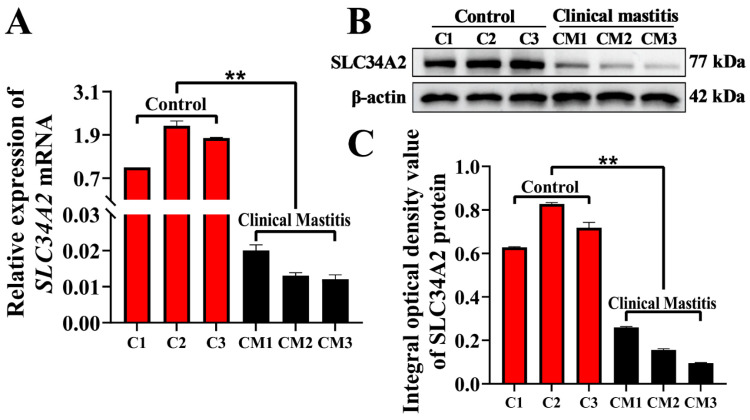
Expression pattern analysis of *SLC34A2* mRNA and protein in MGs. (**A**) mRNA levels of *SLC34A* monitored by qRT-PCR assay. (**B**) Protein bands of *SLC34A* monitored by Western blot assay; the whole Western blots are shown in Figures S1 and S2. (**C**) Optical density of bands. Con/C, control. CM, clinical mastitis. Data are presented as means ± SEM. ** represents *p* < 0.01.

The expression levels of *SLC34A2* mRNA and protein in the MGs of Holstein cows in the Con/C and CM groups were detected using qPCR and Western blotting, respectively. Compared with the Con/C group, the relative expression level of *SLC34A2* mRNA in the CM group was significantly downregulated (*p* < 0.01) (Figure 2A). The *SLC34A2* protein was detected in each sample of the Con/C and CM groups at different expression levels (Figure 2B). The average integrated optical density (IOD) of each band was calculated and used to evaluate SLC34A2 expression. The results showed that the relative expression level of the SLC34A2 protein in the CM group was significantly downregulated compared to that in the Con/C group (*p* < 0.01) (Figure 2C).

### 3.3. Identification of GO Terms and Candidate DEPs Related to SLC34A2

**Figure 3:**
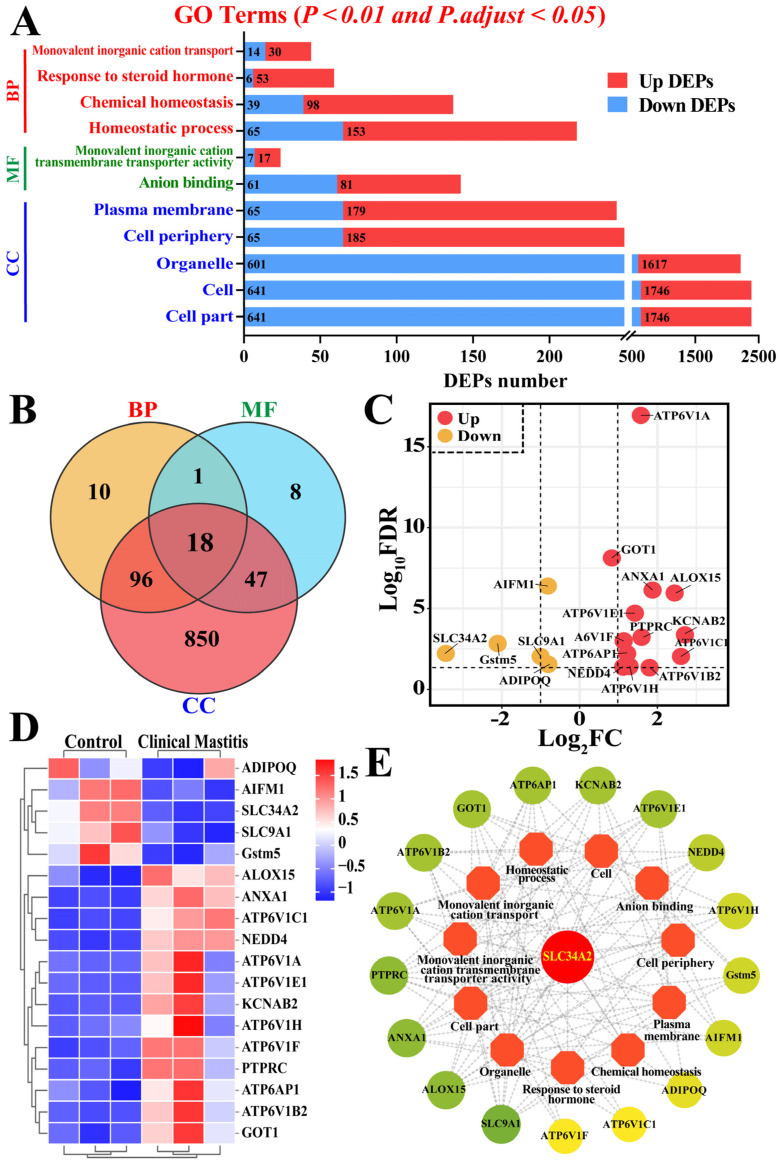
Identification of candidate DEPs and GO terms related to Ca/P metabolism and involved with SLC34A2 between control and clinical mastitis groups based on GO enrichment data. (**A**) Candidate DEPs and GO terms with significance of *p* < 0.01 and *p.adjust* < 0.05 related to Ca/P metabolism and involving SLC34A2. X-axis represents the number of DEPs. Y-axis represents GO terms including biological process (BP), molecular function (MF), and cellular component (CC). (**B**) Venn diagram of candidate DEPs in BP, MF, and CC groups. (**C**) Volcano plots of 18 DEPs, including 5 downregulated and 13 upregulated DEPs. (**D**) Heat map of 18 candidate DEPs. (**E**) PPI network analysis of 18 DEPs and 11 GO terms related to Ca/P metabolism.

GO terms including BP, MF, and CC involved in SLC34A2 were selected to analyze the function and relationships related to Ca/P metabolism and homeostasis in the MGs of the Con/C and CM groups (Figure 3 and Table S1). A total of 125 DEPs from four BP terms, 74 DEPs from two MF terms, and 1011 DEPs from five CC terms were filtered as candidate DEPs (Figure 3A). After overlapping the repeat DEPs using a Venn diagram, the results showed that 18 DEPs shared these 11 GO terms (Figure 3B). A total of 18 DEPs, including 5 downregulated and 13 upregulated DEPs, were identified as crucial candidate DEPs associated with homeostasis, ion binding, or transport (Figure 3C). The heat map showed that these DEPs were significantly differentially expressed in the MGs of the Con/C and CM groups (*p* < 0.01; Figure 3D). The interaction network of the GO terms and candidate DEPs was constructed according to these 18 DEPs, and the results suggested that they interacted directly with the 11 GO terms with at least three or more nodes (Figure 3E). Moreover, the PPI network suggested that SLC34A2 is a crucial candidate DEP that interacts with 11 GO terms and 18 DEPs.

### 3.4. Identification of the Pathways and Candidate DEPs Related to SLC34A2

**Figure 4:**
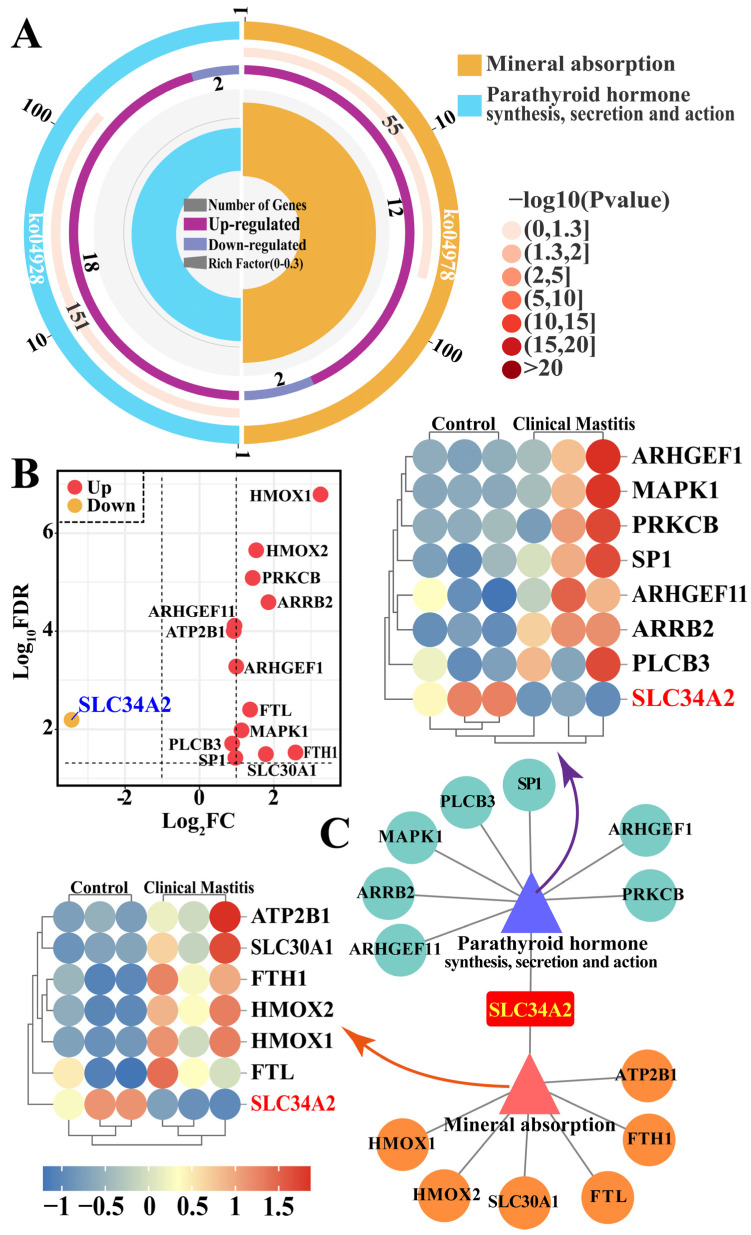
Identification of candidate DEPs and pathways related to Ca/P metabolism and involved with SLC34A2 between the control and clinical mastitis groups based on pathways. (**A**) Candidate DEPs and pathways related to Ca/P metabolism and involving SLC34A2. (**B**) PPI network and heat map analysis of mineral absorption and PTH synthesis, secretion, and action pathways related to Ca/P metabolism involving SLC34A2. (**C**) Volcano plots of 14 DEPs: 1 downregulated and 13 upregulated.

The pathways and candidate DEPs associated with SLC34A2 were selected to identify the potential functions and signal transduction of SLC34A2 associated with Ca/P metabolism and homeostasis in the MGs of Holstein cows with and without CM (Figure 4 and Table S2). Two pathways, mineral absorption (seven DEPs) and parathyroid hormone (PTH) synthesis, secretion, and action (eight DEPs), were identified by DIA proteomic analysis (Figure 4A). A total of 14 unique DEPs, including 13 upregulated and 1 downregulated, were present in these pathways, which shared only SLC34A2 (Figure 4B). The interaction network of the pathways and candidate DEPs was also constructed based on these 14 DEPs; the results suggest that SLC34A2 acts as a bridge between the pathways and DEPs. A heat map shows that these proteins were differentially expressed in the MGs of the Con/C and CM groups (Figure 3C). The results indicate that the 14 DEPs play important roles in Ca/P metabolism and homeostasis and in the development of CM in dairy cows, especially SLC34A2.

### 3.5. Identification of Candidate DEPs Interacting with SLC34A2 Based on GO Terms and Pathways

**Figure 5:**
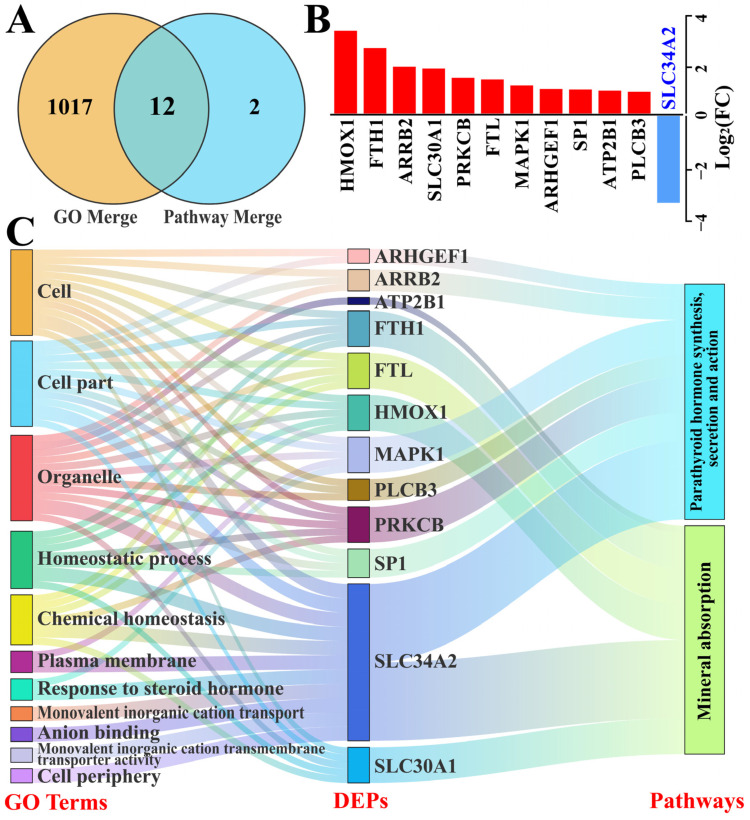
Identification of candidate DEPs related to Ca/P metabolism and involved with SLC34A2 between the control and clinical mastitis groups based on the GO enrichment and pathway data. (**A**) Venn diagram of candidate DEPs in the merged GO terms and pathways. (**B**) Relative expression levels of 12 DEPs quantified by DIA proteomics; the y-axis represents the log2 (FC) values. (**C**) Sankey diagram of the 11 GO terms, 12 shared DEPs, and two pathways related to Ca/P metabolism and ion homeostasis.

We focused on the significant candidate DEPs interacting with SLC34A2 that were associated with Ca/P metabolism and homeostasis, based on GO terms and pathways (Figure 5). A total of 1031 DEPs (Table S3) included in the GO terms and pathways were selected as candidate DEPs, which shared 12 DEPs after overlapping repeated ones (Figure 5A). These DEPs included 11 upregulated and 1 downregulated protein (Figure 5B) and were selected as the core candidate DEPs for further analysis of the functions and relationships among DEPs, GO terms, and pathways using the Sankey diagram. The results show that pathways and biological processes were enriched with these DEPs, particularly SLC34A2 (Figure 5C). These results further confirm that SLC34A2 is involved in Ca/P metabolism and ion homeostasis in the MGs of Holstein cows with CM.

### 3.6. PPI Network Analyses of the Candidate DEPs, GO Terms, and Pathways in Ion Metabolism and Homeostasis

**Figure 6:**
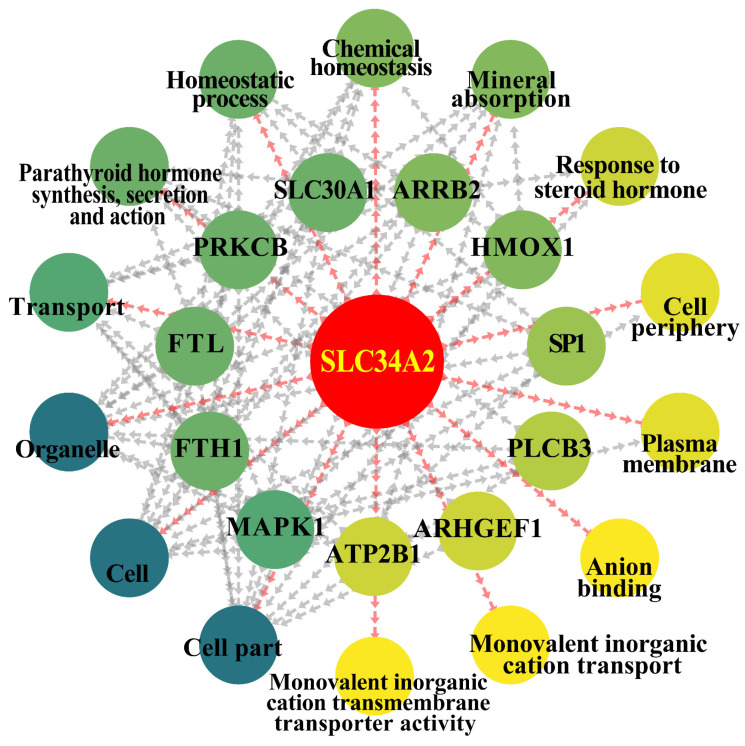
PPI network analyses of the candidate DEPs, GO terms, and pathways in ion metabolism and homeostasis in the MGs between the control and clinical mastitis groups.

According to the results of distribution and expression patterns, GO functional enrichment, and pathway analysis, SLC34A2 is a crucial DEP that may regulate ion metabolism and homeostasis. To confirm this relationship, a PPI network was constructed based on the data (Figure 6). The results demonstrate that SLC34A2 is a unique DEP that interacts with all selected GO terms, pathways, and DEPs. For instance, SLC34A2 may directly regulate ion metabolism, transport, and mineral absorption via heme oxygenase-1 (HMOX1), ferritin heavy chain 1 (FTH1), and specific protein 1 (SP1), respectively. SLC34A2 may also directly regulate homeostatic processes and chemical homeostasis via different proteins, such as protein kinase C beta type (PRKCB) and ferritin light chain (FTL). This evidence confirms that SLC34A2 can directly or indirectly regulate Ca/P metabolism and homeostasis and participate in the occurrence and development of CM in dairy cows.

## 4. Discussion

Ca, which is present mainly as hydroxyapatite in the skeleton or other chemical structures in the extracellular fluid, plasma, and cells [12], plays essential roles in metabolism, blood clotting, enzyme activation, and other physiological or pathological processes in mammals. Additional Ca or P supplementation in lactating dairy cows, particularly during the perinatal period, may cause unbalanced Ca/P metabolism and increase the risk of mastitis. Thus, identifying the potential functional proteins involved in Ca/P transport and further understanding the regulatory and absorptive mechanisms of Ca/P metabolism in the MGs of dairy cows are beneficial for the prevention and treatment of mastitis. In the present study, we found that SLC34A2 plays an important role in Ca/P metabolism and homeostasis, and is negatively correlated with the occurrence and development of clinical mastitis in dairy cows.

The transcellular transport of P and Ca is interlinked with transcellular sodium flux [19] and is associated with the expression of genes mediating transcellular P and Ca transport [20]. SLC34A2 plays an important role in the maintenance of the overall phosphate homeostasis (such as hydroxyapatite, α-glycerophosphate, and β-glycerophosphate) that is essential for proper cellular functions [21]. Research shows that SLC34A2 is expressed in many digestive organs with different expression patterns [21], particularly in the process of intestinal phosphate absorption. In the present study, we first confirmed that the SLC34A2 protein is located mainly in the MECs of MGs, indicating that functions such as Ca/P metabolism and transport of SLC34A2 may be associated with MEC functions. MECs have important effects on milk secretion, inflammation, and immunity [1] and are equally important in ion transport [22]. Previous studies have suggested that the transport and absorption of Ca/P in MECs are necessary to maintain the integrity of the mammary epithelium and MA [23]. Moreover, SLC34A2 has been shown to be abundantly expressed in MGs and positively correlated with milk, protein, lactose, and fat yields in sheep and cattle [24]. Furthermore, the SLC34A2 protein, a proteotypic marker of MEC identity, is correlated with the persistence of mammary ductal structures and identifiable secretory MA structures developed by the mammary epithelium [25]. These results confirm that SLC34A2 affects the morphological structure of the mammary gland via Ca/P metabolism and homeostasis and is negatively correlated with the occurrence and development of CM in dairy cows. Nonetheless, SLC34A2’s function and interactive regulation in Holstein cows with CM remain unclear.

We observed no direct correlation between Ca/P metabolism and steroid hormones; however, steroid hormones are still important for Ca/P transport and homeostasis. For instance, adrenal corticosteroids, estrogens, and calcitriol are steroid hormones that may cause hypercalcemia or hypocalcemia by disrupting the normal regulation of Ca balance [26,27]. In addition, two pathways involving SLC34A2 with no significant differences (*p* > 0.05) were selected based on the pathway analysis. Mineral absorption and PTH metabolism are bridge-connected by SLC34A2, and DEPs with significant differences in these pathways play important roles in Ca/P metabolism and homeostasis. Previous studies [28,29] have demonstrated that pseudohypoparathyroidism caused by hypocalcemia and hyperphosphatemia is due to the resistance of the primary target organ to PTH. Conjoint analysis revealed that the 12 shared DEPs were involved in ion homeostasis and absorption. Previous studies have shown that DEPs, such as SLC34A2 [30,31], PRKCB [32], FTH1 [1], and MAPK1 [33], could be putative biomarkers for mastitis. The PPI network displayed 12 candidate DEPs interacting with all selected GO terms, pathways, and DEPs, especially SLC34A2, indicating that these DEPs participate in the regulation of Ca/P metabolism and homeostasis via different routes. Previous studies have proved that Ca/P intake in birds adapted by increasing the mRNA expression of Ca transporters such as ATP2B1, SLC34A2, and SLC30A1 [34,35,36]. It was also reported that the regulation of mineral homeostasis including Ca/P metabolism was reflected via altered mRNA abundances of FTH1, FTL, and SP1, which were involved in phospholipase C, calcium signaling, nuclear factor of activated T cell signaling, and immunomodulatory implications [37,38].

In summary, we found that SLC34A2 can directly or indirectly regulate Ca/P metabolism and homeostasis and participate in the occurrence and development of clinical mastitis in dairy cows. These findings provide a theoretical basis for the prevention and treatment of CM via the regulation of Ca/P homeostasis. However, the relationships and regulatory mechanisms between SLC34A2 and identified DEPs in Holstein cows with CM are incompletely understood, necessitating further research for comprehensive elucidation.

## 5. Conclusions

IHC and IF staining indicates that SLC34A2 is primarily located in the mammary epithelial cells of healthy Holstein cows or CM. Compared to the Con/C group, the *SLC34A2* mRNA and protein levels in the MGs of the CM group were significantly downregulated. Based on the DIA proteomics data, we identified 12 critical DEPs included in 11 GO terms and two pathways that interacted with SLC34A2. The PPI network demonstrated that SLC34A2 was a unique DEP that interacted with all selected GO terms, pathways, and DEPs. These results revealed that SLC34A2 and DEPs such as HMOX1, PRKCB, and SLC30A1 could regulate Ca/P metabolism and homeostasis via the function of parathyroid hormone and calcitonin, and further participate in the occurrence and development of clinical mastitis in dairy cows, providing a theoretical basis for understanding how SLC34A2 and DEPs functions in Ca/P homeostasis in Holstein cows with CM.

## Data Availability

The data that support the findings of this study are available from the corresponding author upon reasonable request.

## Supplementary Materials

The following supporting information can be downloaded at: https://www.mdpi.com/article/10.3390/ani14091275/s1, Table S1: The DEPs filtered from the GO terms according to the DIA proteomics sequence database; Table S2: The DEPs selected from the KEGG pathways according to the DIA proteomics sequence database; Table S3: The significant candidate DEPs related to Ca/P metabolism and homeostasis based on GO terms and pathways analysis; Figure S1: Whole Western blot of *SLC34A2* (77 kDa); Figure S2: Whole Western blot of β-actin (42 kDa).
