# Supplementary figures and images for: SLC34A2 Targets in Calcium/Phosphorus Homeostasis of Mammary Gland and Involvement in Development of Clinical Mastitis in Dairy Cows
Source: Animals (Basel). 2024 Apr 24;14(9):1275. doi: 10.3390/ani14091275 (PMC11083581; doi:10.3390/ani14091275)

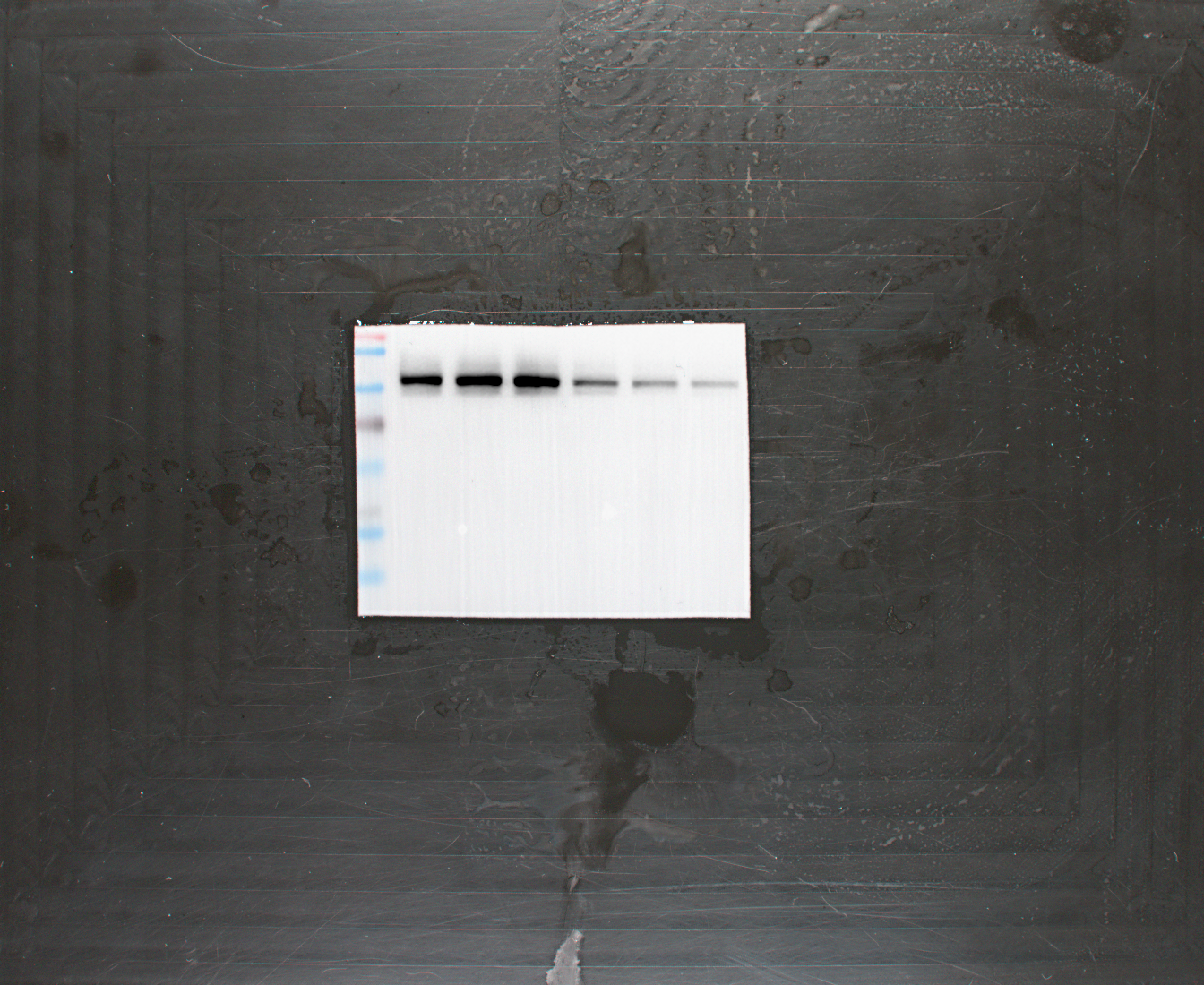

Supplement: Supplementary file 1 [file animals-14-01275-s001.zip › Figure S1 Whole Western blot of SLC34A2 (77 kDa).Tif]

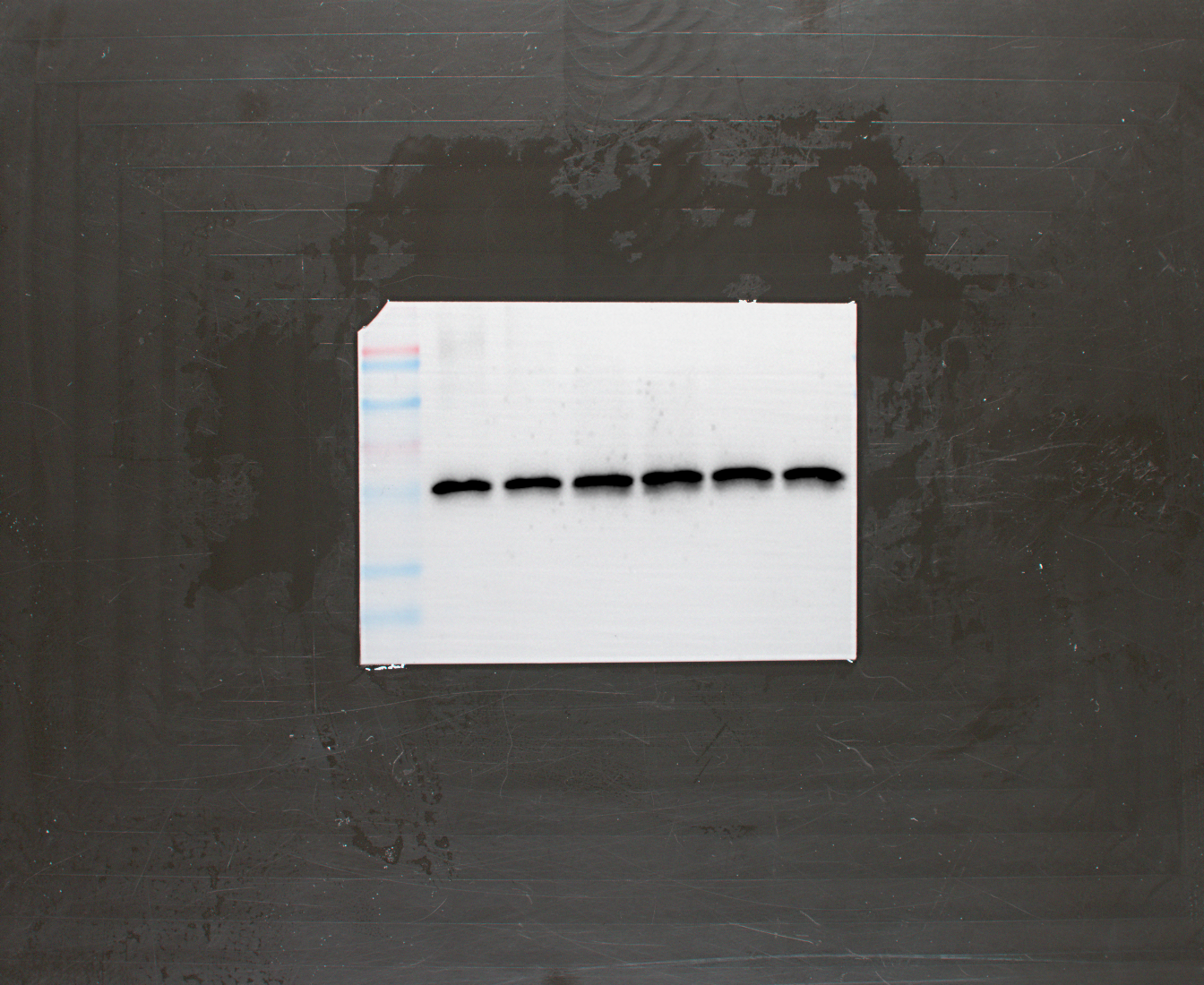

Supplement: Supplementary file 1 [file animals-14-01275-s001.zip › Figure S2 Whole Western blot of a┬-actin (42 kDa).Tif]
